# HMOX1 interacts with BNIP3 to modulate neuronal ferroptosis after spinal cord ischemia-reperfusion injury via a mitophagy-dependent mechanism

**DOI:** 10.1038/s41420-025-02831-z

**Published:** 2025-11-17

**Authors:** Yanni Duan, Yibao Zhang, Fengguang Yang, Mingtao Zhang, Yujun Shi, Zongyan Ma, Xingxing Huang, Wen Li, Yun Lang, Xuchang Hu, Xuewen Kang

**Affiliations:** 1 Department of Neurovascular Function Examination, The Second Hospital of Lanzhou University, Lanzhou, 730000 China; 2 The Second Hospital & Clinical Medical School, Lanzhou University, Lanzhou, 730000 China; 3 Orthopaedics Key Laboratory of Gansu Province, The Second Hospital of Lanzhou University, Lanzhou, 730000 China; 4 Department of Rehabilitation Medicine, The Second Hospital of Lanzhou University, Lanzhou, 730000 China; 5 Department of Orthopedics, The Second Hospital of Lanzhou University, Lanzhou, 730000 China

**Keywords:** Cell death in the nervous system, Mitophagy

## Abstract

Spinal cord ischemia-reperfusion injury (SCIRI) is a severe secondary complication of trauma, spinal cord decompression, and thoracoabdominal aortic surgery. Ferroptosis, a regulated cell death pathway characterized by iron-dependent accumulation of lethal reactive oxygen species and lipid peroxidation, has been implicated in various pathological conditions. However, its precise role and molecular mechanisms in SCIRI remain unclear. In this study, we demonstrated that ferroptosis contributes to the pathophysiology of SCIRI. Heme oxygenase 1 (HMOX1) was upregulated in both SCIRI rats and neuronal cells subjected to hypoxia-reoxygenation. Genetic knockdown of HMOX1 in vivo and in vitro markedly attenuated neuronal ferroptosis, improving neurological function, whereas HMOX1 overexpression reproduced characteristic ferroptotic events in vitro. HMOX1 upregulation appeared to stimulate autophagic flux and induce substantial mitophagy, suggesting a potential mechanistic link between HMOX1 and ferroptosis promotion. Mitophagy reduction diminished HMOX1-mediated ferroptosis, whereas mitophagy induction acted synergistically with HMOX1. HMOX1 physically interacted with BNIP3, triggering excessive mitophagy and subsequent ferroptosis. In this study, we establish ferroptosis as a critical contributor to SCIRI pathogenesis and identify HMOX1 as a central regulator of this process. Furthermore, mitophagy-dependent ferroptosis, mediated by the HMOX1-BNIP3 axis, emerges as a promising therapeutic target for SCIRI intervention.

## Introduction

Spinal cord ischemia-reperfusion injury (SCIRI) is a condition that can cause severe sensory and motor dysfunction [1]. SCIRI frequently occurs as a complication of thoracic and abdominal aortic surgeries and may also be associated with spinal trauma and intradural tumors [2]. SCIRI may cause permanent spinal cord dysfunction, including paraplegia or mortality, without timely intervention [3]. Therefore, identifying modifiable risk factors for spinal cord repair is essential.

Ferroptosis is a non-apoptotic cell death modality characterized by toxic reactive oxygen species (ROS) accumulation and lipid peroxidation [4–6]. Emerging evidence implicates ferroptosis in numerous diseases, particularly as a therapeutic target in cancer and ischemic organ injury [7–9]. Inhibition of ferroptosis in animal models of traumatic spinal cord injury (SCI) enhanced functional recovery [10–12]. However, the role of ferroptosis in SCIRI remains poorly understood.

Mitochondria are critical organelles that maintain cellular homeostasis [13]. Their vulnerability to damage can lead to dysfunction and disruption of cellular homeostasis, requiring prompt repair or elimination [14]. Mitophagy, a selective form of autophagy, provides quality control by eliminating dysfunctional mitochondria [15, 16]. Growing evidence indicates that moderate mitophagy can sequester iron to mitigate ferroptosis, while excessive mitophagy may provide additional iron to exacerbate it [17, 18]. BNIP3 is a hypoxia-induced mitophagy protein that may drive excessive mitophagy and cell death, whereas NIX mainly regulates basal mitophagy [19, 20]. For instance, the lncRNA SNHG14 has been shown to induce excessive mitophagy in HT22 cells via the microRNA-182-5p/BNIP3 axis [21].

Hemoglobin oxygenase 1 (HMOX1) is an inducible enzyme that oxidizes hemoglobin within cells to release biliverdin, carbon monoxide and free ferrous iron (Fe²⁺) [22]. HMOX1 may exert protective effects on cells by converting the pro-oxidant hemoglobin into the antioxidant biliverdin. Alternatively, it may release large amounts of Fe²⁺ directly into the mitochondrial iron pool, thereby catalyzing ROS production, driving lipid peroxidation and inducing ferroptosis. Therefore, HMOX1 acts as a double-edged sword, providing protection or enhancing cellular vulnerability [23–25]. Elevated HMOX1 expression has been linked to neuronal damage and neurodegeneration in Alzheimer’s and Parkinson’s disease [26]. Therefore, the exact protective or damaging role of HMOX1 in SCIRI needs to be further clarified, as this could lead to potential therapeutic strategies for treating SCIRI.

In this study, we found that ferroptosis is closely associated with SCIRI and that HMOX1 plays a key role in regulating ferroptosis. SCIRI-induced HMOX1 upregulation activates excessive mitophagy through interaction with BNIP3, ultimately triggering iron-dependent ferroptosis. Collectively, this study demonstrates that HMOX1 is a critical contributor to SCIRI pathogenesis and may represent a potential therapeutic target.

## Results

### Ferroptosis in the SCIRI rat model with increased HMOX1 expression levels

**Fig. 1:**
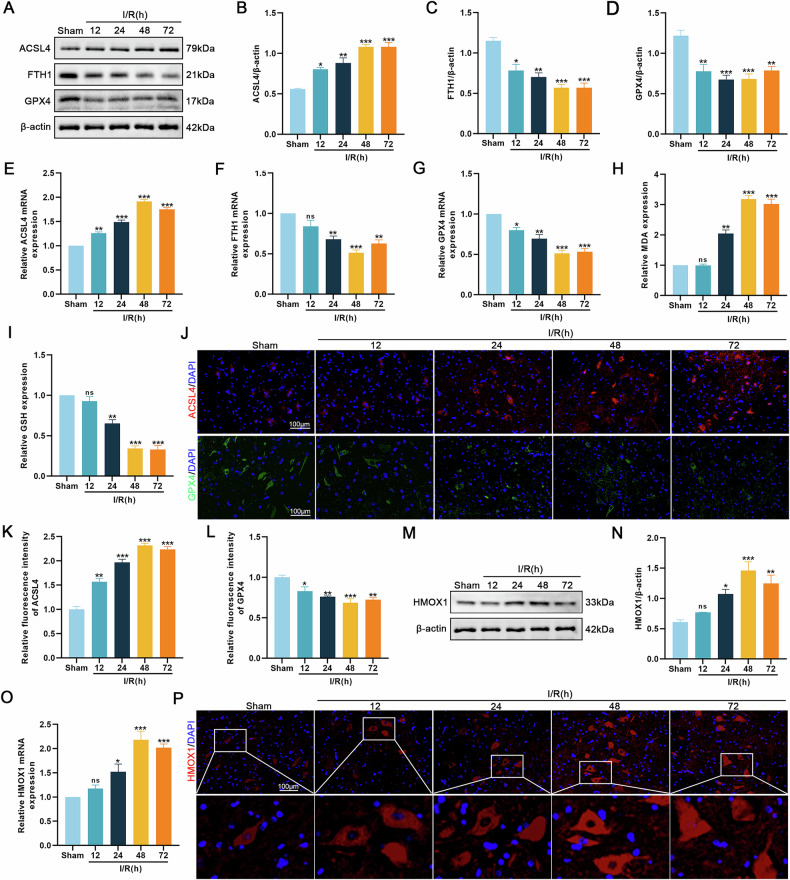
Ferroptosis occurs after SCIRI and HMOX1 expression level is increased. Rat spinal cords were subjected to I/R injury (I: 45 min; R: 12, 24, 48, or 72 h) or sham surgery. **A–D** Western blotting analysis of ferroptosis-related proteins (ACSL4, FTH1, GPX4) in rat spinal cord tissues (*n* = 3). **E–G** qRT-PCR analysis of mRNA expression of ferroptosis-related proteins (*n* = 3). **H**, **I** Determination of relative levels of MDA and GSH (*n* = 6). **J–L** Immunofluorescence staining was used to detect the levels of ACSL4 and GPX4 (*n* = 6). **M**, **O** Detection of HMOX1 protein and mRNA levels after SCIRI using western blotting and qRT-PCR (*n* = 3). **P** Immunofluorescence assay to detect HMOX1 expression after SCIRI (*n* = 6). Compared with the sham group, *P < 0.05, **P < 0.01, ***P < 0.001.

Our bioinformatics analysis on the SCIRI dataset GSE74680 demonstrated that HMOX1 expression was upregulated and associated with ferroptosis in SCIRI (Fig. S1A–D). Ferroptosis is implicated in the pathogenesis of various diseases [27–30]. Initially, we investigated ferroptosis in SCIRI-treated spinal cord tissues. Western blotting revealed that prolonged I/R exposure elevated ACSL4 protein levels while reducing FTH1 and GPX4 expression (Fig. 1A–D). These findings were further supported by qRT-PCR results (Fig. 1E–G). Additionally, extended I/R duration led to increased MDA levels and decreased GSH levels (Fig. 1H, I). Immunofluorescence of ACSL4 and GPX4 yielded results consistent with those of western blotting (Fig. 1J–L). In addition, Both western blotting and qRT-PCR confirmed that HMOX1 protein and mRNA levels increased, reaching their peak at 48 h after I/R induction (Fig. 1M–O). Immunofluorescence analysis of HMOX1 further corroborated these observations (Fig. 1P). In conclusion, our results suggest that HMOX1 may participate in ferroptosis activation following SCIRI.

### Ferroptosis was observed in the OGD/R model, concomitant with increased HMOX1 expression levels

**Fig. 2:**
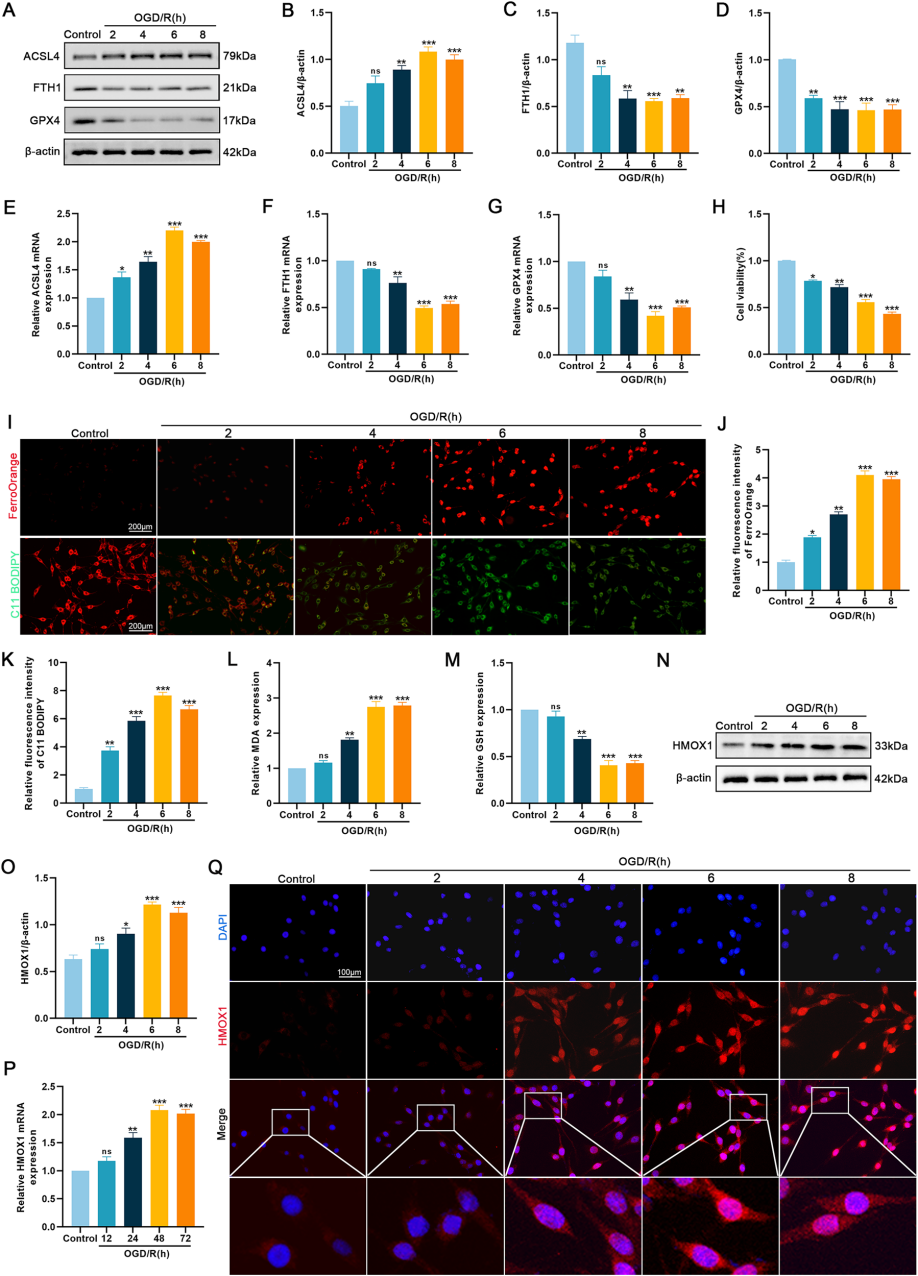
OGD/R treatment induced ferroptosis and increased HMOX1 expression in PC12 cells. PC12 cells were treated with OGD/R (OGD: 2, 4, 6, or 8 h; R: 24 h) or control. **A–D** Western blotting analysis of ferroptosis-related proteins (*n* = 3). **E–G** qRT-PCR analysis of mRNA expression of ferroptosis-related proteins (*n* = 3). **H** CCK-8 assay of cell viability at various OGD/R time points (*n* = 6). **I–K** FerroOrange and C11 BODIPY fluorescent probes measured Fe²⁺ and lipid peroxidation levels (*n* = 6). **L**, **M** The relative values of MDA and GSH concentrations were determined (*n* = 6). **N–P** Western blotting and qRT-PCR analysis of HMOX1 protein and mRNA expression (*n* = 3). **Q** Immunofluorescence analysis of HMOX1 expression (*n* = 6). Compared with the control group, *P < 0.05, **P < 0.01, ***P < 0.001.

PC12 cells were subjected to OGD/R (OGD: 2, 4, 6, or 8 h; R: 24 h). Western blotting revealed that prolonged OGD significantly elevated ACSL4 expression while substantially reducing GPX4 and FTH1 expression (Fig. 2A–D). qRT-PCR yielded consistent results (Fig. 2E–G). The CCK-8 assay results showed a dramatic reduction in cell viability after OGD/R (Fig. 2H). FerroOrange and C11 BODIPY were used to measure the levels of intracellular Fe^2+^ and lipid peroxidation. Both of these levels increased significantly after OGD/R (Fig. 2I–K). We also found that extended OGD/R duration led to considerable elevation in MDA levels while causing marked depletion of GSH (Fig. 2L, M). Collectively, these findings demonstrate that OGD/R treatment induces ferroptosis in neuronal cells.

We further examined the changes in HMOX1 expression following OGD/R treatment. Both the western blotting and qRT-PCR results demonstrated that HMOX1 protein and mRNA levels increased significantly, peaking at 6 h of deprivation followed by 24 h of reoxygenation (Fig. 2N–P). The immunofluorescence results were consistent with these findings (Fig. 2Q). These data indicate that OGD/R injury triggers ferroptosis in PC12 cells, accompanied by upregulation of HMOX1.

### HMOX1 upregulation promotes ferroptosis

**Fig. 3:**
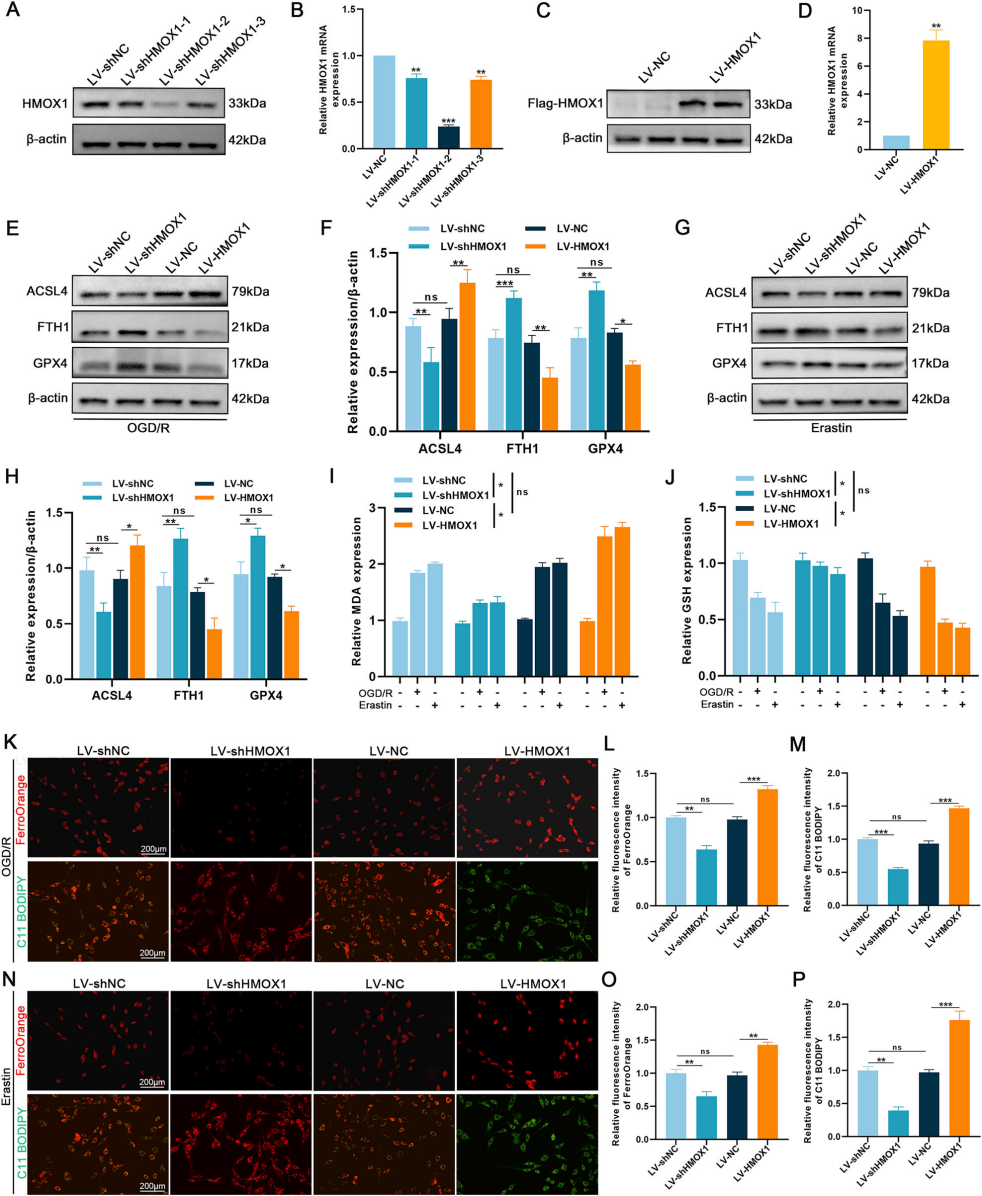
HMOX1 promotes ferroptosis in vitro. PC12 cells were transfected with LV-shHMOX1 or LV-HMOX1. **A**, **B** Western blotting and qRT-PCR analysis of the most effective knockdown sequences (*n* = 3). **C**, **D** Western blotting and qRT-PCR analysis of HMOX1 expression post-LV-HMOX1 transfection (*n* = 3). **E–H** Western blotting analysis of ferroptosis-related protein expression in OGD/R (OGD: 6 h; R: 24 h) and Erastin (2 μM, 24 h) treated cells (*n* = 3). **I**, **J** Quantification of MDA and GSH levels in OGD/R and Erastin treated cells (*n* = 6). **K–P** FerroOrange and C11-BODIPY staining were used to detect the levels of intracellular Fe²⁺ and lipid peroxidation (*n* = 6). *P < 0.05, **P < 0.01, ***P < 0.001.

We pretreated PC12 cells with LV-shHMOX1 or LV-HMOX1 to determine whether HMOX1 is directly involved in inducing ferroptosis. First, we screened the LV-shHMOX1-2 as the most effective knockout sequence (Fig. 3A, B). Similarly, the efficiency of HMOX1 overexpression was evaluated (Fig. 3C, D**)**. Following treatment with OGD/R (OGD: 6 h; R: 24 h) and Erastin (2 μM; 24 h), western blotting revealed that HMOX1 knockdown decreased ACSL4 expression while increasing GPX4 and FTH1 expression, whereas HMOX1 overexpression produced the opposite effect (Fig. 3E–H). Similarly, HMOX1 knockdown decreased MDA levels and increased GSH levels, whereas HMOX1 overexpression showed the opposite trend (Fig. 3I, J). Additionally, FerroOrange and C11 BODIPY staining demonstrated that HMOX1 overexpression exacerbated ferroptosis, whereas HMOX1 knockdown reversed these effects (Fig. 3K–P). These results indicate that HMOX1 promotes ferroptosis in PC12 cells.

### HMOX1 interacts with BNIP3

**Fig. 4:**
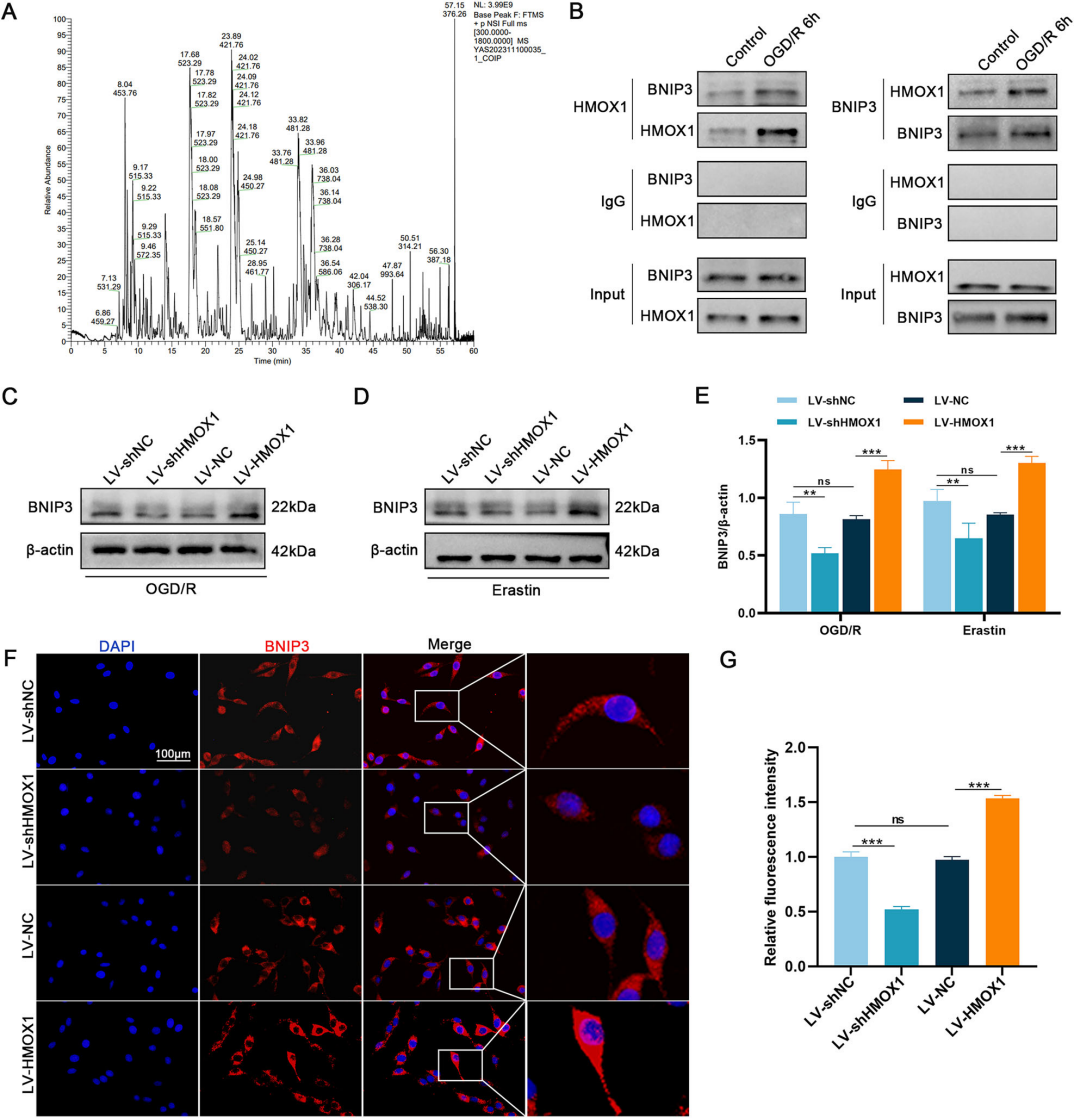
HMOX1 and BNIP3 interactions. **A** IP/MS analysis identified HMOX1-interacting proteins. BNIP3 was identified as an HMOX1-interacting protein. **B** Co-IP assessed endogenous protein interactions in PC12 cell lysates under physiological conditions or post-OGD/R (OGD: 6 h; R: 24 h). Immunoprecipitation with anti-HMOX1 or anti-IgG antibodies, followed by immunoblotting with anti-BNIP3, was performed. Conversely, immunoprecipitation with anti-BNIP3 or anti-IgG, followed by immunoblotting with anti-HMOX1, confirmed endogenous interactions (*n* = 3). **C–E** Western blotting analysis of BNIP3 expression in OGD/R (OGD: 6 h; R: 24 h) and Erastin (2 μM, 24 h) treated cells (*n* = 3). **F**, **G** Immunofluorescence analysis of BNIP3 expression post-HMOX1 knockdown and overexpression (*n* = 6). *P < 0.05, **P < 0.01, ***P < 0.001.

To investigate the potential mechanism by which HMOX1 regulates ferroptosis, we employed IP/MS to identify proteins that interact with HMOX1 during ferroptosis. The IP/MS results revealed that BNIP3 binds to HMOX1 (Fig. 4A). To validate these findings, we performed Co-IP analysis, demonstrating that HMOX1 interacts with BNIP3 under both physiological conditions and OGD/R (OGD: 6 h; R: 24 h) treatment, with enhanced interaction observed after OGD/R. Reverse Co-IP analysis further confirmed that HMOX1 significantly precipitates BNIP3 (Fig. 4B). Subsequent western blotting showed that HMOX1 knockdown reduced BNIP3 expression, whereas HMOX1 overexpression increased BNIP3 expression (Fig. 4C–E). Immunofluorescence results corroborated the western blot data (Fig. 4F, G).

### HMOX1-induced ferroptosis is associated with mitophagy activation

**Fig. 5:**
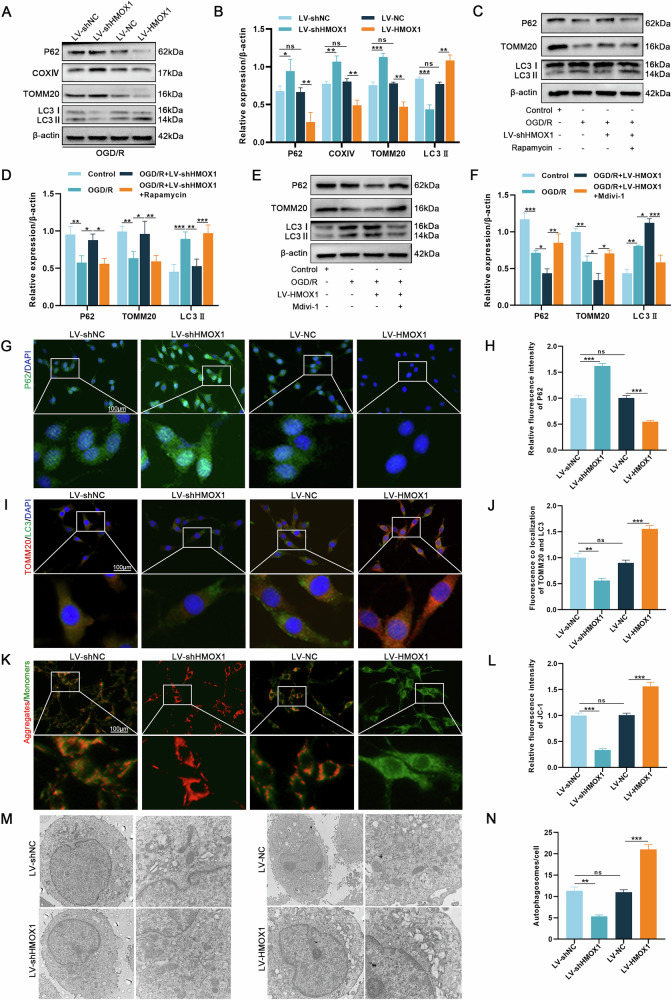
HMOX1 is associated with excessive activation of mitophagy. **A, B** Western blotting analysis of mitophagy-related proteins (P62, COXIV, TOMM20, LC3) in OGD/R-treated cells (OGD: 6 h; R: 24 h) post-LV-shHMOX1 and LV-HMOX1 transfection (*n* = 3). **C–F** Western blotting analysis of mitophagy-related protein expression post-HMOX1 knockdown or overexpression with rapamycin or mdivi-1 intervention (*n* = 3). **G–J** Immunofluorescence staining validated P62 expression and TOMM20/LC3 co-localization (*n* = 6). **K**, **L** JC-1 staining analysis of changes in MMP across groups (*n* = 6). **M**, **N** Transmission electron microscopy analysis of autophagosome numbers across groups (*n* = 6). *P < 0.05, **P < 0.01, ***P < 0.001.

BNIP3, a key mitophagy receptor, regulates intracellular iron levels and is implicated in osteosarcoma ferroptosis [31]. Therefore, we investigated whether HMOX1-induced ferroptosis is linked to mitophagy. Western blotting revealed that HMOX1 knockdown increased P62, TOMM20, and COXIV expression while reducing LC3II levels. Conversely, HMOX1 overexpression produced the opposite effects (Fig. 5A, B). Rapamycin reversed the LV-shHMOX1-mediated effects, and mdivi-1 counteracted the LV-HMOX1-induced changes (Fig. 5C–F).

Next, we assessed mitophagy flux. P62 immunofluorescence and TOMM20/LC3 co-staining results aligned with the immunoblotting data (Fig. 5G–J). MMP measurements showed a substantial decrease following HMOX1 overexpression, whereas HMOX1 knockdown restored MMP (Fig. 5K, L). Finally, transmission electron microscopy confirmed that HMOX1 knockdown reduced autophagosome formation, while HMOX1 overexpression increased it (Fig. 5M, N). These findings support the hypothesis that HMOX1 and BNIP3 interact to trigger the activation of excessive mitophagy.

### BNIP3 overexpression reverses ferroptosis following HMOX1 knockdown

**Fig. 6:**
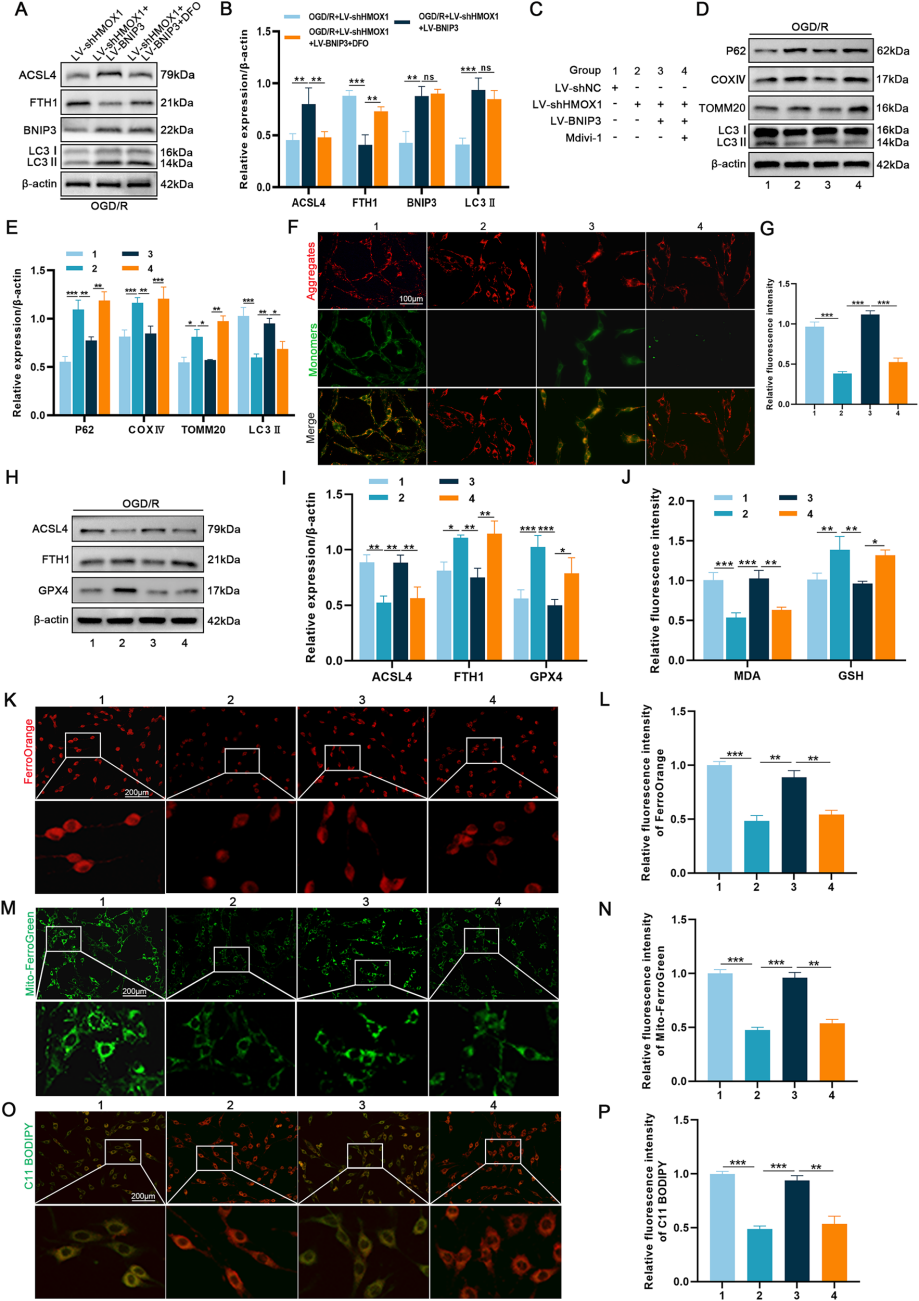
HMOX1 interacts with BNIP3 to regulate ferroptosis through the process of mitophagy. PC12 cells were transfected with LV-BNIP3, which was based on LV-shHMOX1, and then treated with either the iron chelator DFO or the mitophagy inhibitor mdivi-1. **A**, **B** The expression of ACSL4, FTH1, BNIP3, and LC3 was analysed using western blotting after DFO intervention (*n* = 3). **C** Detailed information on the experimental group in this part of the experiment. **D, E** Western blotting analysis of mitophagy-related proteins (*n* = 3). **F**, **G** JC-1 staining assessed MMP changes (*n* = 6). **H**, **I** Western blotting analysis of ferroptosis-related proteins (*n* = 3). **J** Quantification of MDA and GSH levels in OGD/R-treated PC12 cells (*n* = 6). **K–P** FerroOrange, Mito-FerroGreen, and C11 BODIPY probes were used to assess intracellular and mitochondrial Fe²⁺ levels and lipid peroxidation levels (*n* = 6). *P < 0.05, **P < 0.01, ***P < 0.001.

To validate the HMOX1-BNIP3 relationship and BNIP3’s role in ferroptosis, we overexpressed BNIP3 following HMOX1 knockdown. First, PC12 cells from the OGD/R + LV-shHMOX1+LV-BNIP3 group were treated with DFO. Compared with the OGD/R + LV-shHMOX1+LV-BNIP3 group, DFO treatment reduced ACSL4 expression and increased FTH1 expression; meanwhile, BNIP3 and LC3II expression remained unchanged. These results suggest that mitochondria drive ferroptosis by releasing iron in the OGD/R + LV-shHMOX1+LV-BNIP3 group (Fig. 6A, B). Next, mdivi-1 was administered as a mitophagy inhibitor following BNIP3 overexpression after HMOX1 knockdown. Figure 6C provides detailed information about the experimental group. Western blotting revealed that BNIP3 overexpression in HMOX1 knockdown cells significantly decreased P62, COXIV, and TOMM20 and increased LC3II compared to HMOX1 knockdown alone, effects reversed by mdivi-1 (Fig. 6D, E). Subsequently, JC-1 staining results demonstrated that BNIP3 overexpression abolished the elevated MMP caused by HMOX1 knockdown, while mdivi-1 intervention restored MMP level (Fig. 6F, G). These findings suggest that BNIP3 interacts with HMOX1 to regulate mitophagy.

Next, we investigated the changes in ferroptosis. Western blotting revealed that the OGD/R + LV-shHMOX1+LV-BNIP3 group exhibited upregulated ACSL4 expression and downregulated FTH1 and GPX4 expression, which was counteracted by mdivi-1 treatment (Fig. 6H, I). Similarly, LV-shHMOX1+LV-BNIP3 treatment elevated MDA levels and reduced GSH levels, both of which were normalized by mdivi-1 administration (Fig. 6J). Mito-FerroGreen is a fluorescent probe designed specifically to detect Fe^2+^ in mitochondria. FerroOrange, Mito-FerroGreen, and C11 BODIPY staining were consistent with the above findings. Specifically, the overexpression of BNIP3 following HMOX1 knockdown increased mitochondrial Fe^2+^ levels and induced lipid peroxidation. This effect could be reversed by mdivi-1 (Fig. 6K–P). These findings demonstrate that BNIP3-mediated mitophagy contributes significantly to HMOX1-induced ferroptosis in neuronal cells.

### HMOX1 knockdown promotes neurological recovery in rats after SCIRI

**Fig. 7:**
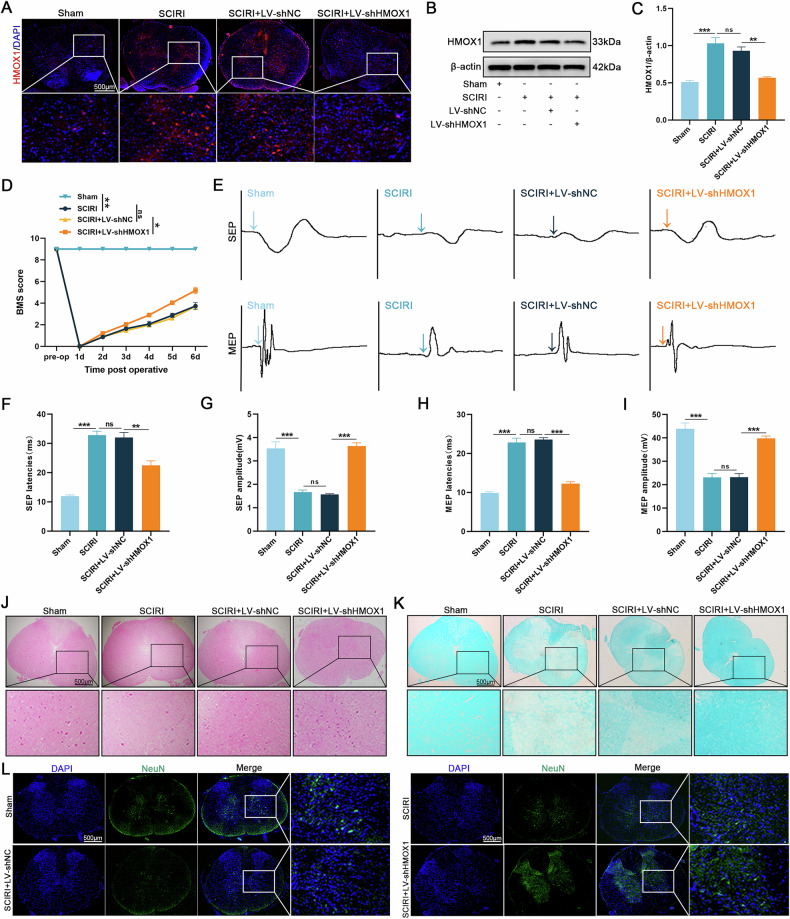
HMOX1 knockdown promotes neurological function recovery after SCIRI. **A** Immunofluorescence staining of HMOX1 expression in spinal cord tissues post-SCIRI (I: 45 min; R: 48 h) (*n* = 6). **B**, **C** Western blot analysis of HMOX1 expression across groups (*n* = 3). **D** Results of BMS scores of rats after SCIRI (*n* = 6). **E** Neurophysiological evaluation via SEP and MEP (arrow indicates evoked potential onset) (*n* = 6). **F–I** Quantitative analysis of SEP and MEP latency and amplitude (*n* = 6). **J** Representative HE staining of spinal cord tissues across groups post-SCIRI (*n* = 6). **K** Representative LFB staining of spinal cord tissues across groups post-SCIRI (*n* = 6). **L** Representative immunofluorescence staining of NeuN in spinal cord tissues across groups post-SCIRI (*n* = 6). *P < 0.05, **P < 0.01, ***P < 0.001.

The therapeutic impact of intravertebral LV-shHMOX1 injection was investigated to assess the neuroprotective effects of HMOX1 knockdown in SCIRI rats. Immunofluorescence revealed significantly reduced HMOX1 expression at the injury site in the SCIRI + LV-shHMOX1 group, consistent with western blotting results (Fig. 7A–C). Behavioral analysis using BMS scores demonstrated significantly higher scores in the SCIRI + LV-shHMOX1 group than in the SCIRI + LV-shNC group (Fig. 7D). SEP and MEP monitoring confirmed that HMOX1 knockdown enhanced sensory and motor function recovery post-SCIRI (Fig. 7E–I). HE and LFB staining assessed the impact of HMOX1 knockdown on spinal cord tissue at the injury site, revealing intact tissue, orderly nerve fibers, and regular myelin sheath morphology in the sham group. In contrast, the SCIRI group exhibited disrupted spinal cord structure, neuronal damage, and pronounced demyelination. Compared to the SCIRI + LV-shNC group, the SCIRI + LV-shHMOX1 group showed significantly reduced neuronal damage, histological changes, and demyelination (Fig. 7J, K). Immunofluorescence revealed significantly reduced NeuN expression in the anterior horn after SCIRI, whereas HMOX1 knockdown significantly increased NeuN expression (Fig. 7L). These findings indicate that HMOX1 knockdown mitigates spinal cord tissue damage and enhances sensory and motor function recovery in SCIRI rats.

### HMOX1 knockdown inhibits ferroptosis and excessive mitophagy in SCIRI rats

**Fig. 8:**
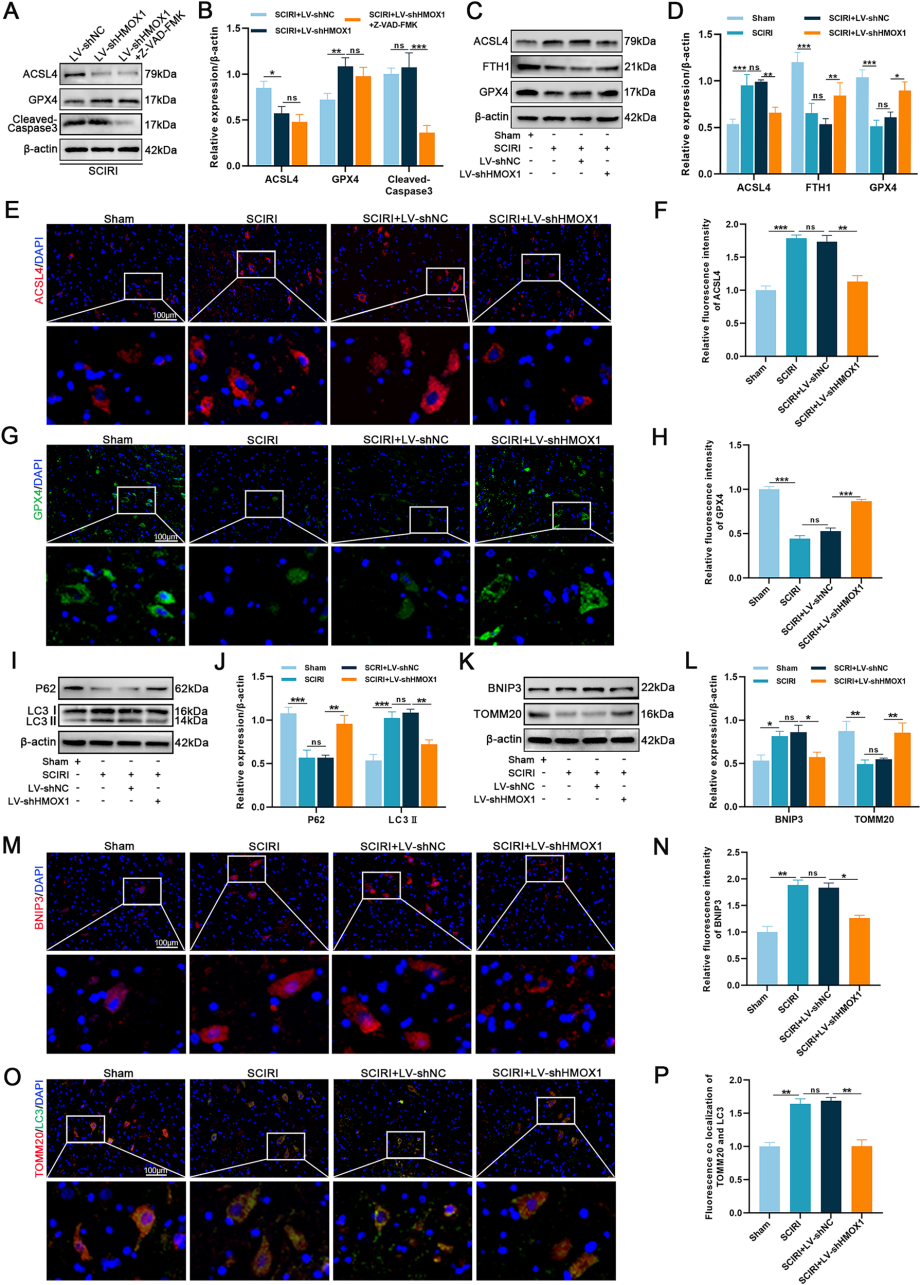
HMOX1 knockdown inhibits ferroptosis and excessive mitophagy in SCIRI rats. **A**, **B** Z-VAD-FMK intervention was administered to rats with HMOX1 knockdown, and the expression of ACSL4, GPX4, and cleaved caspase 3 was analyzed by western blotting (*n* = 3). **C**, **D** Western blotting analysis of ferroptosis-related protein expression (*n* = 3). **E–H** Immunofluorescence staining validated ACSL4 and GPX4 expression (*n* = 6). **I–L** Western blotting analysis of mitophagy-related protein expression (*n* = 3). **M–P** Immunofluorescence staining assessed BNIP3 expression and TOMM20/LC3 co-localization (*n* = 6). *P < 0.05, **P < 0.01, ***P < 0.001.

The effects of HMOX1 knockdown on ferroptosis and mitophagy were examined in SCIRI rats. First, Z-VAD-FMK intervention was administered to rats in the SCIRI + LV-shHMOX1 group. Cleaved caspase-3 expression reduced significantly, while ACSL4 and GPX4 levels remained unchanged. This suggests that HMOX1-induced ferroptosis in the SCIRI model occurs independently of apoptosis (Fig. 8A, B). Western blotting revealed reduced ACSL4 and increased FTH1 and GPX4 expression in the SCIRI + LV-shHMOX1 group compared with the SCIRI + LV-shNC group (Fig. 8C, D). Immunofluorescence staining of ACSL4 and GPX4 corroborated western blotting findings (Fig. 8E–H). Additionally, western blotting revealed that HMOX1 knockdown increased P62 and TOMM20 expression while reducing BNIP3 and LC3II expression (Fig. 8I–L). BNIP3 immunofluorescence and TOMM20/LC3 co-localization staining confirmed these findings (Fig. 8M–P). These findings demonstrate that HMOX1 knockdown suppresses ferroptosis and excessive mitophagy in SCIRI rats.

## Discussion

In this study, we establish that ferroptosis contributes to the pathogenesis of SCIRI and, for the first time, demonstrate that HMOX1 knockdown suppresses ferroptosis and excessive mitophagy, thereby promoting neurological recovery in rats. HMOX1 was found to interact with BNIP3, modulating the mitophagy pathway, promoting excessive mitophagy activation, and leading to ferroptosis. Thus, HMOX1 has been proposed as a potential therapeutic target for SCIRI.

HMOX1 primarily exerts protective effects on cells [32]. However, emerging evidence suggests that excessive HMOX1 expression may be detrimental [24, 25, 33]. The overexpression of HMOX1 increases the levels of unstable intracellular Fe²⁺, thereby promoting ferroptosis [34, 35]. Bioinformatics analysis revealed elevated HMOX1 expression and ferroptosis in SCIRI. We confirmed the expression of ferroptosis-related proteins and HMOX1 in vivo, as well as the occurrence of ferroptosis and markedly elevated HMOX1 expression following SCIRI. Immunofluorescence revealed predominant HMOX1 expression in neuronal cells, indicating its role in neuronal damage. To further elucidate the role of HMOX1 in ferroptosis during SCIRI, we employed HMOX1 knockdown in vivo, effectively inhibited ferroptosis and promoted neurological function recovery in rats following SCIRI. These findings suggest that HMOX1 promotes ferroptosis and impairs spinal cord function recovery in SCIRI rats.

To elucidate the mechanism of HMOX1-induced ferroptosis, we identified BNIP3 as a novel interacting protein using IP/MS and Co-IP techniques. Immunoblotting and immunofluorescence analyses demonstrated that HMOX1 modulates BNIP3 expression, with HMOX1 knockdown reducing BNIP3 levels and HMOX1 overexpression producing the opposite effect, indicating a positive correlation between HMOX1 and BNIP3. As a critical mitophagy receptor, BNIP3 may selectively induce excessive mitophagy, resulting in cell death [20]. Thus, the interaction between HMOX1 and BNIP3 may constitute a pivotal mitophagy regulatory mechanism.

Mitophagy is a critical mechanism that regulates mitochondrial function and cellular homeostasis [15]. Recent studies have established a strong correlation between ferroptosis and mitophagy [18, 36]. Mitophagy sequesters iron within mitochondrial autophagosomes, under mild stress or early iron overload conditions, thereby attenuating ferroptosis. However, excessive activation of mitophagy may provide additional iron, thereby aggravating ferroptosis [17]. We revealed that mitophagy was enhanced following HMOX1 upregulation, with the opposite effect observed upon HMOX1 downregulation, while mdivi-1 and rapamycin reversed these effects. Thus, we propose a novel mechanism by which HMOX1 induces ferroptosis. To further investigate whether mitophagy triggered during HMOX1-induced ferroptosis is associated with BNIP3, BNIP3 was overexpressed in the context of HMOX1 knockdown. The results revealed that HMOX1-deficient neurons exhibited enhanced mitophagy and ferroptosis following BNIP3 overexpression, while mdivi-1 reversed these effects. This finding further supports the hypothesis that HMOX1 induces ferroptosis by excessively activating mitophagy.

Although these findings indicate that HMOX1 interacts with BNIP3 to induce mitochondrial dysfunction and ferroptosis in PC12 cells through excessive mitophagy activation, HMOX1 knockdown improved sensorimotor function recovery and suppressed ferroptosis and excessive mitophagy in SCIRI rats in vivo. However, several limitations and challenges should be noted. First, clinical specimen was hindered by the unavailability of human spinal cord tissue samples. Second, the in vitro OGD/R model could not fully reproduce the SCIRI-associated complex microenvironmental changes. Further research is needed to clarify the precise mechanisms underlying the HMOX1-BNIP3 interaction and its regulation of excessive mitophagy activation.

In conclusion, we established that HMOX1 plays a critical role in neuronal pathology following SCIRI. Ferroptosis represents a key injury mechanism in SCIRI and a promising therapeutic target. The interaction between HMOX1 and BNIP3 constitutes a pivotal molecular event that promotes cellular ferroptosis and induces excessive mitophagy. A deeper understanding of HMOX1-related molecular mechanisms and signaling pathways may facilitate the development of novel diagnostic and therapeutic strategies for the regulation of neuronal survival and death following SCIRI. This underscores the potential of targeting HMOX1 as a viable therapeutic strategy for SCIRI, offering novel insights for its treatment.

## Materials and methods

### SCIRI model

Adult female Sprague-Dawley rats (200-250 g) were provided by the Experimental Animal Center of Lanzhou University. The study was approved and overseen by the Ethics Committee of Lanzhou University (approval number: D2024-289). All surgical procedures were performed under sterile conditions. Anesthesia was induced by intraperitoneal pentobarbital injection (40 mg/kg). First, the left femoral vein and right femoral artery were isolated and cannulated for fluid return, blood withdrawal, and blood pressure monitoring. Next, a 1.0 cm segment of the spinal cord centered at T10 was exposed through layered dissection. Hemorrhage was induced by withdrawing 30% of the total blood volume (duration: 10 min, rate: 0.5 ml/min). The blood volume withdrawn (ml) was calculated as the body mass (g)×7.4%×30%. Blood was collected and stored in a heparinized sterile syringe at 4°C. Resuscitation was initiated after 45 min of hemorrhage. Somatosensory evoked potential (SEP) and motor evoked potential (MEP) were monitored throughout the experimental procedure. Manual bladder expression was performed every 8 h postoperatively until reflex bladder emptying returned.

In the sham group, the wound was closed directly following inguinal vascular isolation and removal of the T10 spinous process and lamina to expose the dura mater. In the SCIRI + LV-shNC, SCIRI + LN-shHMOX1, and SCIRI + LN-shHMOX1+Z-VAD-FMK groups, the respective lentiviral vectors and Z-VAD-FMK (10 μM; Selleckchem) were administered intrathecally after SCIRI. Spinal cord tissues were harvested 48 h after surgery for subsequent experiments.

### Functional behavior analysis

Rats were maintained on a 12-h light-dark cycle with ad libitum access to food and water. All rats were acclimated in the testing chamber or apparatus for 1 h before behavioral testing. Neurological function was scored using the Basso Mouse Scale (BMS) at predetermined time points, beginning on day 1 after injury.

### Tissue preparation, hematoxylin-eosin, and luxol fast blue staining

Forty-eight hours after surgery, rats were anesthetized with 4% pentobarbital sodium and underwent systemic perfusion with phosphate-buffered saline (PBS) and 4% paraformaldehyde. The isolated spinal cord tissues were fixed in 4% paraformaldehyde for 24 h, followed by ethanol gradient dehydration and paraffin embedding. Sections (5 μm) were prepared for hematoxylin-eosin (HE) and luxol fast blue (LFB) staining (Solarbio).

### PC12 cell culture

PC12 cells (Shanghai Institute of Biochemistry and Cell Biology, Shanghai, China) were routinely cultured in high-glucose Dulbecco’s modified Eagle’s medium (DMEM; Meilun Biotech) supplemented with 10% fetal bovine serum in an incubator at 37°C with 5% CO₂. Cells were cultured for 24 h with or without the ferroptosis inducer Erastin (2 μM; PeproTech), the mitophagy inducer rapamycin (80 nM; Selleckchem), the mitophagy inhibitor mdivi-1 (40 μM; Selleckchem), and the iron chelating agent deferoxamine (DFO; 10 μM; Selleckchem) for 24 h. Cells were collected when confluence reached 70%–80%.

### Lentivirus transfection

When the PC12 cell density reached approximately 30% in culture flasks, lentivirus (GeneChem) was transfected at a multiplicity of infection of 100. The medium was replaced 48 h after transfection. HMOX1 and BNIP3 expression levels were quantified using qRT-PCR and western blotting.

### OGD/R model

An OGD/R model was created to simulate in vitro SCIRI using established protocols [37]. PC12 culture medium was replaced with glucose-free DMEM, and the cells were subjected to OGD in a tri-gas incubator (94% N_2_, 5% CO_2_, 1% O_2_; 37°C) for 2, 4, 6, or 8 h. Subsequently, the medium was replaced with high-glucose DMEM, and the cells were transferred to a regular incubator (5% CO_2_; 37°C) for 24 h of reoxygenation. The control group cells were cultured in high-glucose in a regular incubator for the same duration.

### Cell viability, malondialdehyde, and glutathione assays

Cell viability and the levels of malondialdehyde (MDA) and glutathione (GSH) in tissues or cell lysates were evaluated using a Cell Counting Kit-8 (CCK-8) assay (Dojindo), MDA assay kit (Beyotime), and GSH assay kit (Beyotime). The absorbance was measured using a BioTek microplate reader (Agilent Technologies).

### JC-1 staining

Changes in mitochondrial membrane potential (MMP) changes were assessed using the JC-1 MMP Assay Kit (Beyotime). Cells were incubated with JC-1 staining solution at 37°C for 20 min in a cell culture incubator, rinsed twice with JC-1 staining buffer, and imaged using a fluorescence microscope (Olympus).

### FerroOrange, Mito-FerroGreen, and C11 BODIPY staining

Intracellular and mitochondrial Fe²⁺ and lipid peroxidation levels in live cells were detected using FerroOrange (Dojindo), Mito-FerroGreen (Dojindo), and C11 BODIPY (Thermo Fisher Scientific) fluorescence probes. Cells were seeded on coverslips, treated, and stained according to the manufacturer’s instructions. Subsequently, cells were imaged using a fluorescence microscope (Olympus).

### qRT-PCR

Total RNA was extracted using TRIzol (Takara) and reverse-transcribed using the PrimeScript RT Master Mix Kit (Takara). HMOX1, ACSL4, FTH1, and GPX4 expression levels were quantified using the SYBR PrimeScript RT-PCR Kit (Takara), and target mRNA expression levels were normalized to GAPDH. Relative expression levels were calculated using the 2^−ΔΔCT^ method. The primers used for qRT-PCR are listed in Table S1.

### Western blot analysis

Total proteins were extracted from PC12 cells and rat spinal cord tissues using RIPA lysis buffer (Beyotime). Protein concentrations were measured using a BCA Protein Assay Kit (Beyotime) and analyzed by SDS-PAGE. Antibodies used in this study were HMOX1 (1:2000, Abcam), ACSL4, FTH1, GPX4, LC3, COXIV, TOMM20, and cleaved-caspase 3 (all 1:1000, Abmart), BNIP3 (1:1000, Cell Signaling Technology), P62 (1:5000, Abmart), Flag-Tag (1:2000, Cell Signaling Technology), and β-actin (1:1000, Beyotime).

### Immunofluorescence

Paraffin sections were dewaxed, hydrated, and antigen retrieved. Cells were fixed in 4% paraformaldehyde for 30 min and permeabilized with 0.2% Triton X-100 for 15 min. The subsequent staining procedures were identical for both tissue and cell samples. After blocking with 10% goat serum at 37°C for 1 h, the samples were incubated with primary antibodies at 4°C overnight. After washing, the samples were incubated with fluorescent secondary antibodies at 37°C for 1 h. Nuclei were stained with DAPI, and imaged using a fluorescence microscope (Olympus).

### Immunoprecipitation and co-immunoprecipitation

PC12 cells were washed twice with PBS, harvested, and lysed with IP binding buffer and protease inhibitor (Beyotime). An aliquot was reserved for western blotting, while the remaining lysate was incubated with specific antibodies and protein A/G beads using a BeaverBeads Protein A/G Immunoprecipitation (IP) Kit (Beaver Bioscience), gently mixed to ensure homogeneous bead-antibody complexes, and incubated overnight at 4 °C on a rotator. Magnetic beads were separated using a magnetic stand. Thereafter, beads were boiled in sodium dodecyl sulfate loading buffer and analyzed by western blotting.

### IP coupled with mass spectrometry (IP/MS)

Total protein was extracted from PC12 cells, and IP was performed as described using specific antibodies and protein A/G-agarose beads, followed by MS (Thermo Fisher) analysis of the isolated immunoprecipitates.

### Statistical analysis

Statistical analysis was conducted using GraphPad Prism (version 9.0), with all experiments performed in at least triplicate. The results are presented as the mean ± standard deviation. Student’s t-test was employed for two-group comparisons, while one-way analysis of variance was used for multi-group analyses. *P* < 0.05 was considered statistically significant.

## Supplementary information

Supplementary Information Legends

Figure S1

Table S1

Original Western Blots

## Data Availability

All data are available from the corresponding author upon reasonable request.
