# Supplementary material for: HMOX1 interacts with BNIP3 to modulate neuronal ferroptosis after spinal cord ischemia-reperfusion injury via a mitophagy-dependent mechanism
Source: Cell Death Discov. 2025 Nov 17;11:536. doi: 10.1038/s41420-025-02831-z (PMC12623955; doi:10.1038/s41420-025-02831-z)
Supplement: Supplementary file 1 — Supplementary Information Legends [file 41420_2025_2831_MOESM1_ESM.docx]

**Supplementary Information Legends**

**Supplementary Figure S1 Bioinformatics analysis. A** box plot of the samples' expression levels following normalization with the GSE74680 dataset. **B** Following standardization, the GSE74680 dataset was subjected to principal component analysis (PCA). **C** Volcano plot of DEG. Red signifies up-regulation, blue shows down-regulation, and gray represents residuals. **D** Visualization of GO enrichment findings that include HMOX1. *P<0.05, **P<0.01, ***P<0.001.

**Supplementary Table S1.** Sequences of primers used for quantitative real-time PCR.
