# Supplementary figures and images for: HMOX1 interacts with BNIP3 to modulate neuronal ferroptosis after spinal cord ischemia-reperfusion injury via a mitophagy-dependent mechanism
Source: Cell Death Discov. 2025 Nov 17;11:536. doi: 10.1038/s41420-025-02831-z (PMC12623955; doi:10.1038/s41420-025-02831-z)

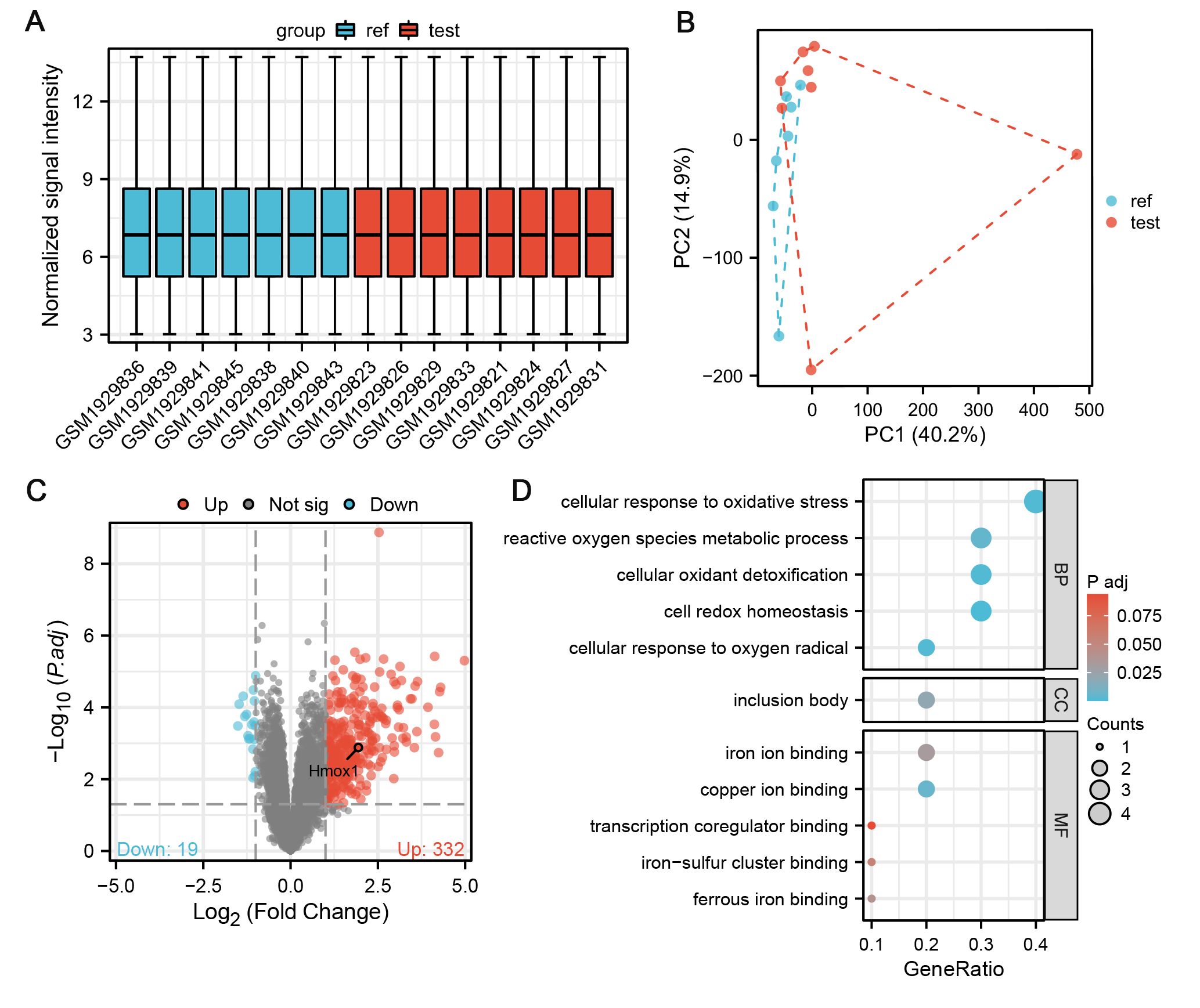

Supplement: Supplementary file 2 — Figure S1 [file 41420_2025_2831_MOESM2_ESM.tif]
