# Supplementary material for: HMOX1 interacts with BNIP3 to modulate neuronal ferroptosis after spinal cord ischemia-reperfusion injury via a mitophagy-dependent mechanism
Source: Cell Death Discov. 2025 Nov 17;11:536. doi: 10.1038/s41420-025-02831-z (PMC12623955; doi:10.1038/s41420-025-02831-z)
Supplement: Supplementary file 3 — Table S1 [file 41420_2025_2831_MOESM3_ESM.docx]

Table S1. Sequences of primers used for quantitative real-time PCR

| Gene | Forward (5–3′) | Reverse (5–3′) |
| --- | --- | --- |
| HMOX1 | AGGTGTCCAGGGAAGGCTTTAA | GCATAGACTGGGTTCTGCTTGTT |
| ACSL4 | AAGCAGCAAGTGGATAGCAGTTA | CAGTAACGGAAGCAGCAGTAAGA |
| FTH1 | GTTGTATGCCTCCTACGTCTATCT | GGTTCTGCAGCTTCATCAGTTTC |
| GPX4 | CCGGCTACAATGTCAGGTTTGA | CCGGCTACAATGTCAGGTTTGA |
| GAPDH | CAGCCTCAAGATCATCAGCA | ATGATGTTCTGGAGAGCCCC |
