# Supplementary material for: HMOX1 interacts with BNIP3 to modulate neuronal ferroptosis after spinal cord ischemia-reperfusion injury via a mitophagy-dependent mechanism
Source: Cell Death Discov. 2025 Nov 17;11:536. doi: 10.1038/s41420-025-02831-z (PMC12623955; doi:10.1038/s41420-025-02831-z)
Supplement: Supplementary file 4 — Original Western Blots [file 41420_2025_2831_MOESM4_ESM.docx]

**Original western blots**

Fig. 1A


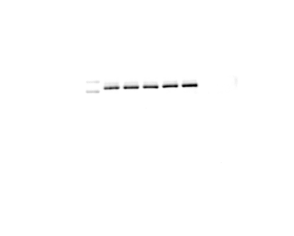

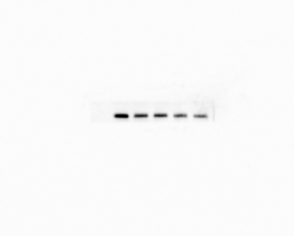

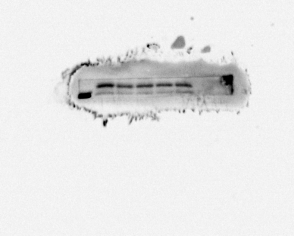

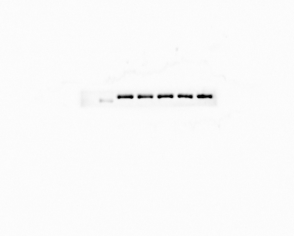


**β-actin**

**GPX4**

**FTH1**

**ACSL4**

Fig. 1M


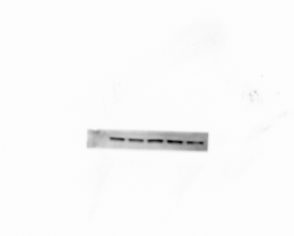

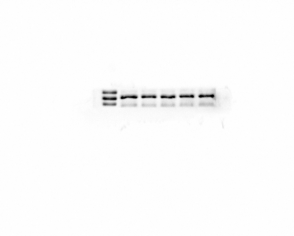


**β-actin**

**HMOX1**

Fig. 2A


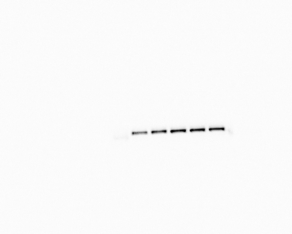

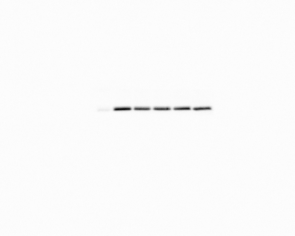

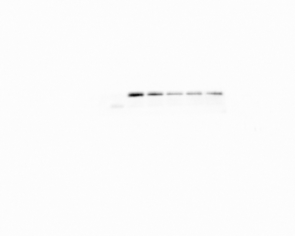

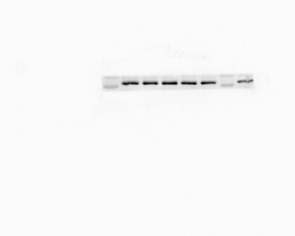


**ACSL4**

**FTH1**

**GPX4**

**β-actin**

Fig. 2N


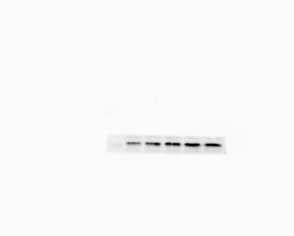

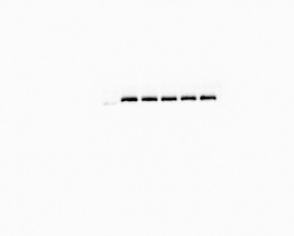


**β-actin**

**HMOX1**

Fig. 3A


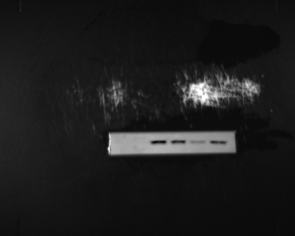

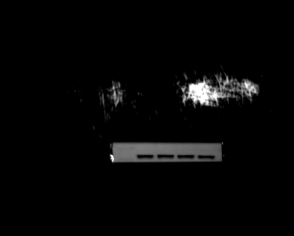


**β-actin**

**HMOX1**

Fig. 3C


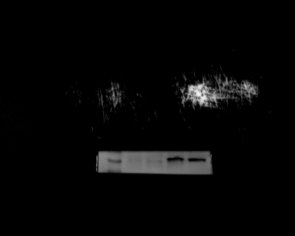

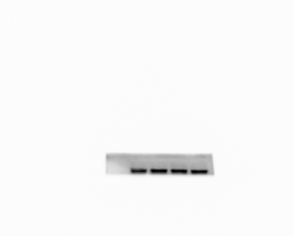


**Flag-HMOX1**

**β-actin**

Fig. 3E


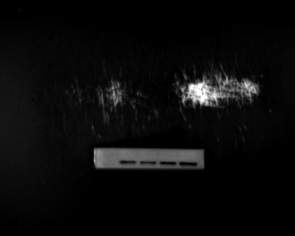

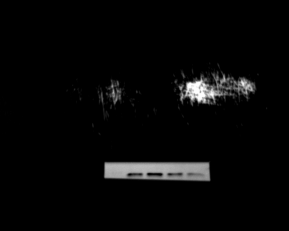

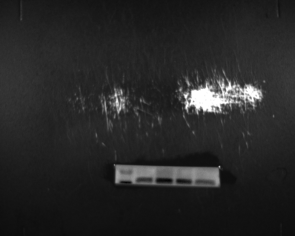

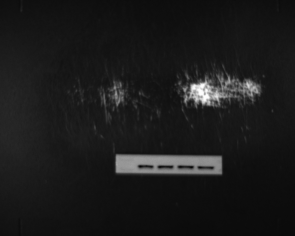


**β-actin**

**GPX4**

**ACSL4**

**FTH1**

Fig. 3G


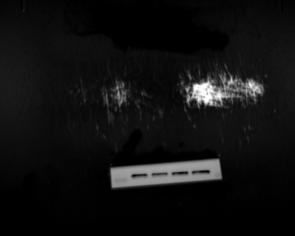

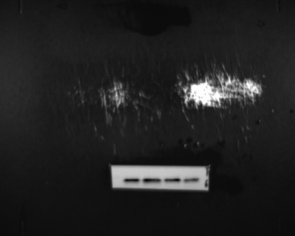

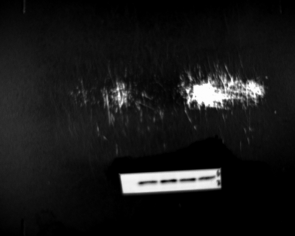

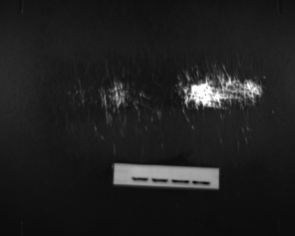


**β-actin**

**GPX4**

**FTH1**

**ACSL4**

Fig. 4B


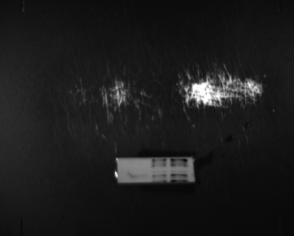

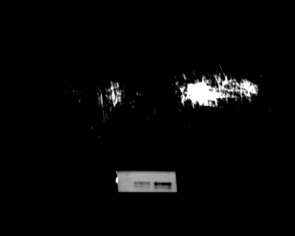

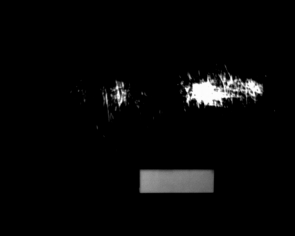

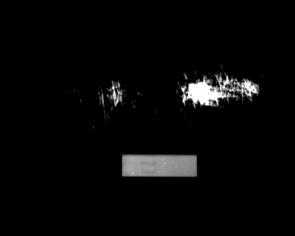


**HMOX1(IgG)**

**BNIP3(IgG)**

**HMOX1(HMOX1)**

**BNIP3(HMOX1)**


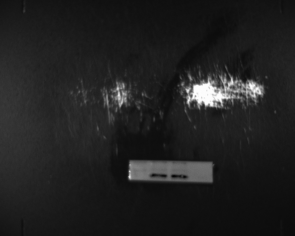

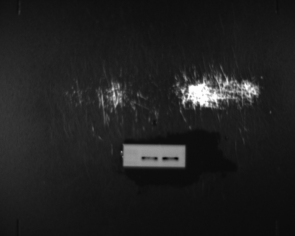


**HMOX1(Input)**

**BNIP3(Input)**


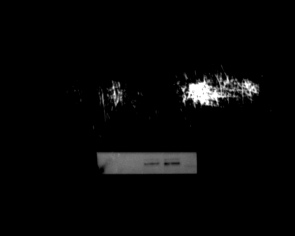

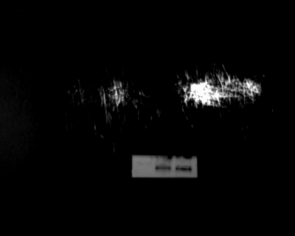

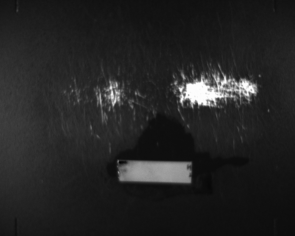

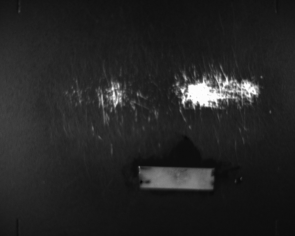


**BNIP3(IgG)**

**HMOX1(IgG)**

**BNIP3(BNIP3)**

**HMOX1(BNIP3)**


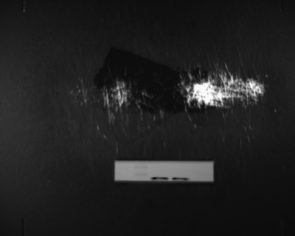

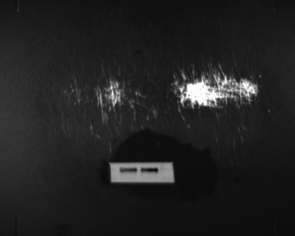


**BNIP3(Input)**

**HMOX1(Input)**

Fig. 4C


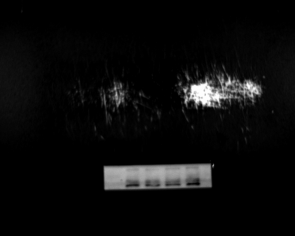

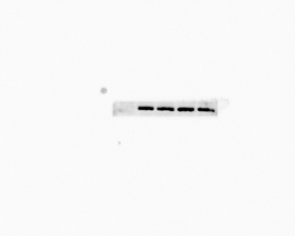


**β-actin**

**BNIP3**

Fig. 4D


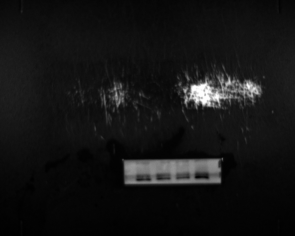

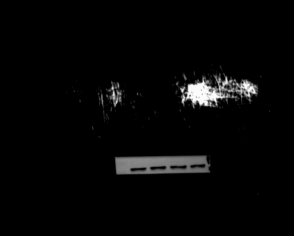


**β-actin**

**BNIP3**

Fig. 5A


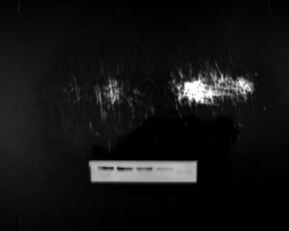

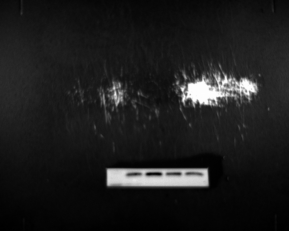

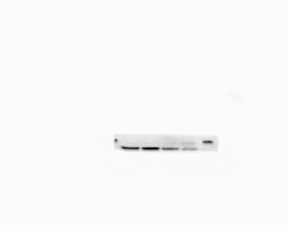

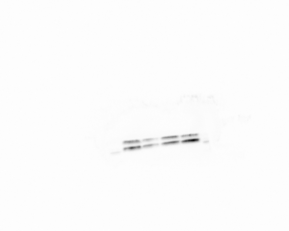


**LC3**

**TOMM20**

**COXⅣ**

**P62**


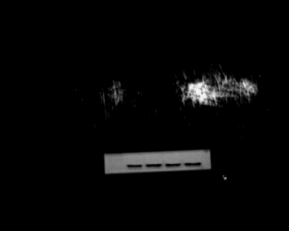


**β-actin**

Fig. 5C


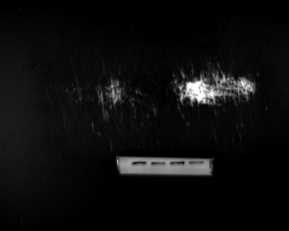

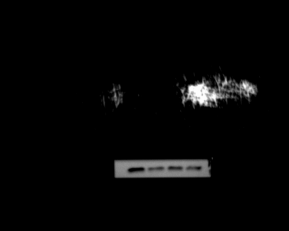

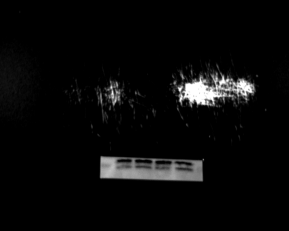

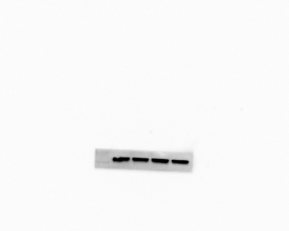


**β-actin**

**LC3**

**P62**

**TOMM20**

Fig. 5E


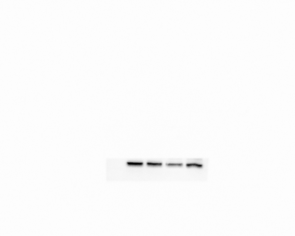

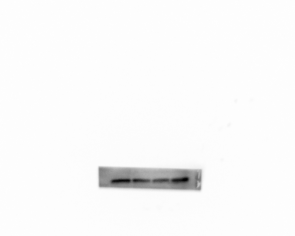

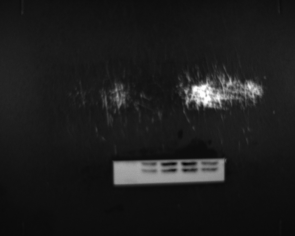

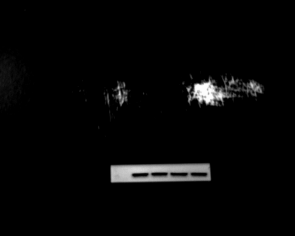


**β-actin**

**LC3**

**TOMM20**

**P62**

Fig. 6A


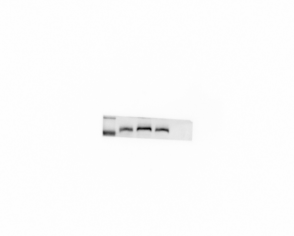

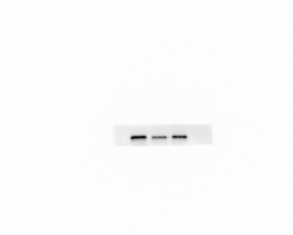

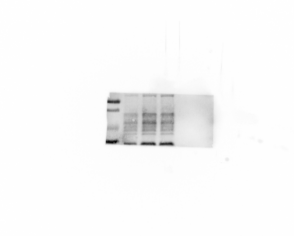

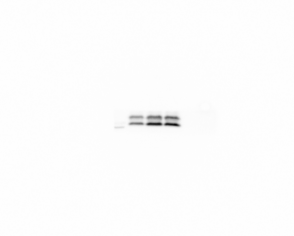

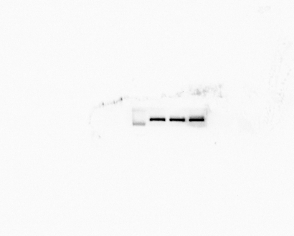


**LC3**

**BNIP3**

**FTH1**

**β-actin**

**ACSL4**

Fig. 6D


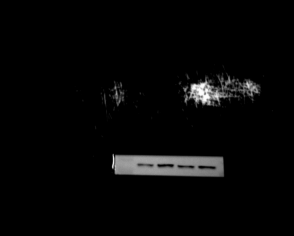

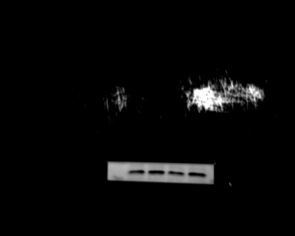

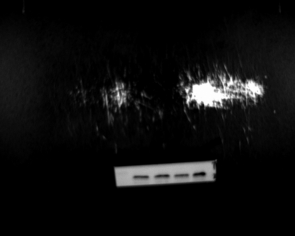

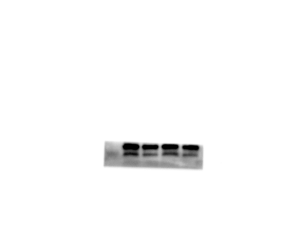

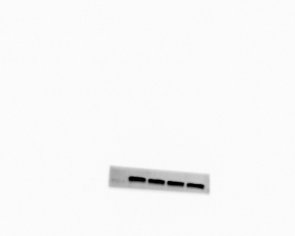


**β-actin**

**LC3**

**TOMM20**

**COXⅣ**

**P62**

Fig. 6H


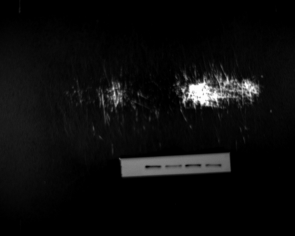

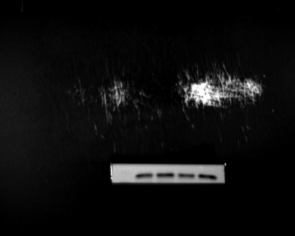

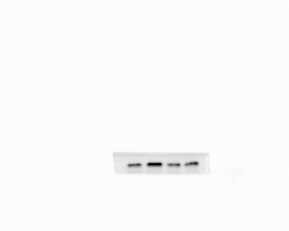

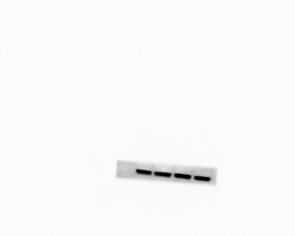


**β-actin**

**GPX4X4**

**FTH1**

**ACSL4**

Fig. 7B


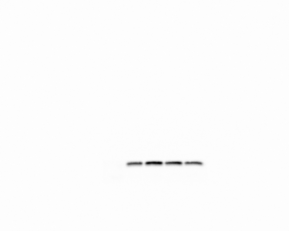

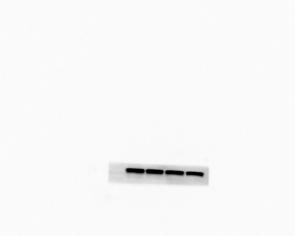


**β-actin**

**HMOX1**

Fig. 8A


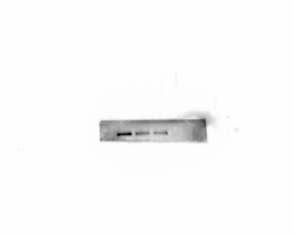

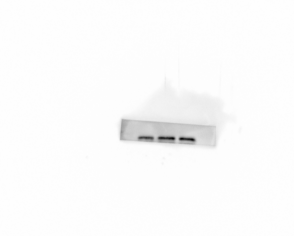

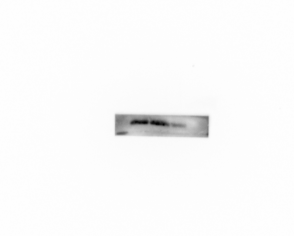

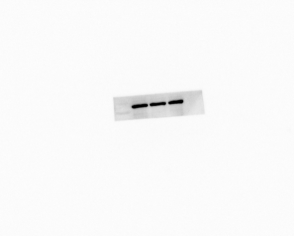


**ACSL4**

**β-actin**

**Cleaved Caspase3**

**GPX4**

Fig. 8C


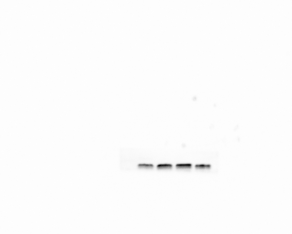

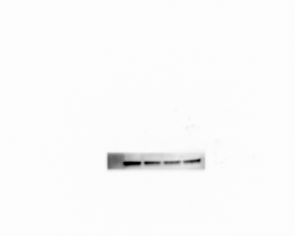

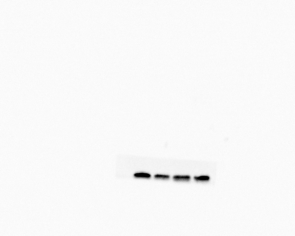

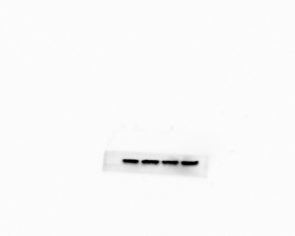


**β-actin**

**GPX4**

**FTH1**

**ACSL4**

Fig. 8I


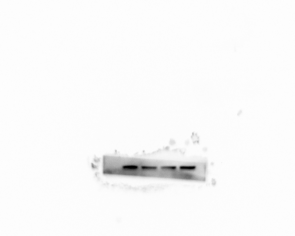

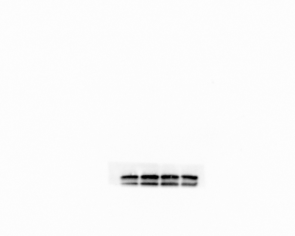

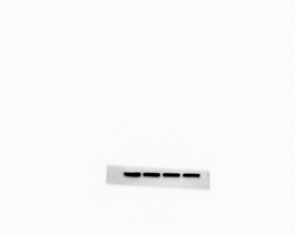


**β-actin**

**LC3**

**P62**

Fig. 8K


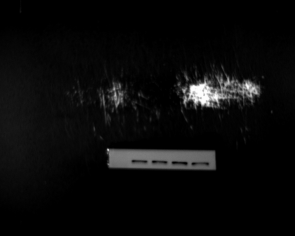

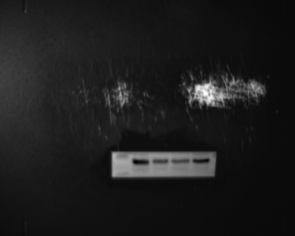

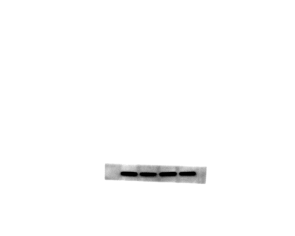


**β-actin**

**TOMM20**

**BNIP3**
